# Influence of Oxygen Vacancy Behaviors in Cooling Process on Semiconductor Gas Sensors: A Numerical Analysis †

**DOI:** 10.3390/s18113929

**Published:** 2018-11-14

**Authors:** Jianqiao Liu, Wanqiu Wang, Zhaoxia Zhai, Guohua Jin, Yuzhen Chen, Wusong Hong, Liting Wu, Fengjiao Gao

**Affiliations:** 1 College of Information Science and Technology, Dalian Maritime University, Dalian 116026, China; 18940855382@163.com (W.W.); shirlyllei@dlmu.edu.cn (Z.Z.); jingh@dlmu.edu.cn (G.J.); hongwusong@outlook.com (W.H.); wuliting0125@outlook.com (L.W.); gaofj11@outlook.com (F.G.); 2 Department of Material Science and Engineering, Dalian Maritime University, Dalian 116026, China; cyz7817@dlmu.edu.cn

**Keywords:** semiconductor, gas sensor, oxygen vacancy, diffusion equation, numerical analysis

## Abstract

The influence of oxygen vacancy behaviors during a cooling process in semiconductor gas sensors is discussed by the numerical analysis method based on the gradient-distributed oxygen vacancy model. A diffusion equation is established to describe the behaviors of oxygen vacancies, which follows the effects of diffusion and exclusion in the cooling process. Numerical analysis is introduced to find the accurate solutions of the diffusion equation. The solutions illustrate the oxygen vacancy distribution profiles, which are dependent on the cooling rate as well as the temperature interval of the cooling process. The gas-sensing characteristics of reduced resistance and response are calculated. Both of them, together with oxygen vacancy distribution, show the grain size effects and the re-annealing effect. It is found that the properties of gas sensors can be controlled or adjusted by the designed cooling process. The proposed model provides a possibility for sensor characteristics simulations, which may be beneficial for the design of gas sensors. A quantitative interpretation on the gas-sensing mechanism of semiconductors has been contributed.

## 1. Introduction

The successful invention of ZnO gas sensors by Seiyama in 1962 started the new era of the application of semiconductor in a gas detecting field [1]. From then on, several kinds of semiconductors were put into practice to develop advanced gas sensors, which contained a variety of sensing materials such as SnO_2_ [2,3,4], ZnO [5,6], and WO_3_ [7] as well as in different forms such as bulk [8], thick film [9,10], and thin film [11,12,13]. Recently, semiconductor quantum dots were also introduced into the investigation of gas sensors [14,15,16].

The gas-sensing mechanism of semiconductors was understood by Morrison who concluded that gas detection was completed by the change in resistance of a sensor [17]. A specific region near the surface of a semiconductor grain, which is usually called a depletion layer, would decrease its resistance when exposed to reducing gases while the resistance would increase if oxidizing gases were introduced. This transducing process was achieved by the reaction between reducing gas and adsorbed oxygen or the competitive adsorption between adsorbed oxygen and oxidizing gas [18]. In 2009, Yamazoe concluded the gas-sensing mechanism of semiconductor into three levels including the receptor function, the transducer function, and the utility factor [19]. Among these levels, the receptor function was the most complex one and described how a single semiconductor grain responded to a stimulant gas. It included several decisive factors such as the grain size and shape [20,21,22], surface composite [23,24], the type of adsorbed oxygen [25,26], and the characteristics of oxygen vacancies [27,28].

The oxygen vacancy (*V_O_*) plays a vital role because it ionizes and provides semi-conductive nature to the metal-oxide system [29], which would be insulative without any defect inside. The behaviors of oxygen vacancies are essential questions about the gas-sensing mechanism of semiconductors. The formation of *V_O_* usually takes place during sintering in which the oxygen atom escapes from the crystal lattice according to Equation (1).

(1) $$O_{O}^{\times }↔V_{O}^{\times }+\frac{1}{2}O_{2}$$

Then, some of *V_O_* are involved into the grain, which grows up during the sintering. The *V_O_* on the grain surface act as adsorption site of adsorbed oxygen while the ones inside the grain provide quasi-free electrons for conductance after their ionization. Furthermore, the defects would migrate in the grain if the temperature is above the absolute zero K especially when the operating temperature of a semiconductor gas sensor is usually above 200 °C. The importance of *V_O_* was recognized and there were some specific studies on it [30,31,32]. However, in the theoretical investigations, the roles of *V_O_* were simplified since its density had to be assumed as a uniform distribution throughout the grain [17,18]. This assumption was good for calculation, but it deviates from the actual condition. Therefore, a gradient-distributed oxygen vacancy model was proposed based on the experimental influences of the cooling rate on the gas-sensing characteristics of SnO_2_ thin films [33,34].

The migration of oxygen vacancies in a semiconductor grain is concluded as diffusion and exclusion. The former one results from the density gradient of defects in the grain, which follows Fick’s second law. The latter one is an excluding tendency, which is provided by the cooling process because the crystal lattice is stable if it is free of defects [35,36]. Thus, the diffusion equation of oxygen vacancies in the cooling process is established, as seen in Equation (2).

(2) $$\frac{\partial N_{V}(r,t)}{\partial t}=D_{V}(t)\frac{\partial^{2}N_{V}(r,t)}{\partial r^{2}}-P(t)N_{V}(r,t)$$

**Figure 1:**
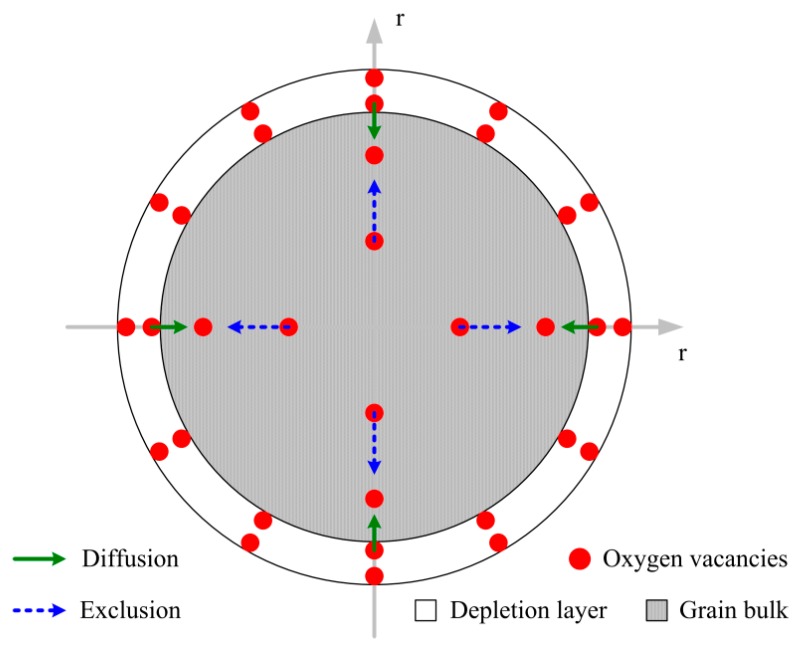
Schematic drawing of the migration of oxygen vacancies under the diffusion and exclusion effects in a sphere coordinates established in an ideal grain.

In this scenario, *N_V_*(*r*,*t*) represents the density of oxygen vacancies at a place of *r* and time of *t* in an ideal spherical grain in which the sphere coordinates are established as Figure 1. Considering the symmetry of sphere coordinates, only one-dimensional model is applied.

The first term of the right side of the diffusion equation indicates the diffusion effect, which is expressed by Fick’s second law. *D_V_*(*t*) is the temperature-dependent diffusion coefficient and it is formulated as Equation (3).

(3) $$D_{V}(t)=D_{0}\exp(-\frac{E_{D}}{kT})=D_{0}\exp[-\frac{E_{D}}{k(T_{0}-\beta t)}]$$

In this case, *D*_0_ is the pre-exponent constant and *E_D_* is activation energy of diffusion. *k* is the Boltzmann constant. *T*_0_ is the initial temperature when the cooling process starts. *β* is the cooling rate and *t* is the time elapsed in the cooling process. The other term of the right side of the diffusion equation denotes the exclusion effect. *P* is the possibility of the exclusion for a defect moving outwards to a nearby position in unit time and it can be formulated as Equation (4).

(4) $$P(t)=\nu_{0}\exp(-\frac{E_{\varphi }-E_{0}}{kT})=\nu_{0}\exp[-\frac{E_{\varphi }-E_{0}}{k(T_{0}-\beta t)}]$$

In this case, *ν*_0_ is the thermal vibration frequency of the oxygen atom. *E_φ_* represents the activated energy of defect migration and *E*_0_ is the unit energy decrease of the system, which resulted from the one-step exclusion of defects. Therefore, the diffusion equation of Equation (2) can be rewritten as Equation (5).

(5) $$\frac{\partial N_{V}(r,t)}{\partial t}=D_{0}\exp[-\frac{E_{D}}{k(T_{0}-\beta t)}]\frac{\partial^{2}N_{V}(r,t)}{\partial r^{2}}-\nu_{0}\exp[-\frac{E_{\varphi }-E_{0}}{k(T_{0}-\beta t)}]N_{V}(r,t)$$

The analytical solution of the diffusion equation is not available. Thus, several presumptions had to be made in the previous works [31,33,34] to simplify the calculation, which simulated the *V_O_* distribution and gas-sensing characteristics of the semiconductor grain. However, some of the presumptions were far from the practical situations, which leads to an inaccuracy in the solutions that describe the distributions of oxygen vacancies.

In the present work, an attempt on finding the accurate solutions is made by using numerical analysis methods. The numerical solutions that follow the diffusion equation of Equation (5) are found to describe the kinetics of oxygen vacancies and their time-dependent distributions. Furthermore, they are used to evaluate the electrical characteristics of semiconductor gas sensors such as resistance and response to reducing gases. The influences of the grain size effects, the re-annealing effect, and the controlled temperature interval on the sensor properties are also discussed.

## 2. Methods

The computational tool of MATLAB is used to find the numerical solutions of the diffusion equation of Equation (5). A discrete *N_V_*(*r*,*t*) is established in the two-dimensional array of *N_V_*(*i*,*j*), which derives from *N_V_*(*r*,*t*) = *N_V_*(*i*Δ*r*,*j*Δ*t*). In this scenario, Δ*r* and Δ*t* are the infinite small fragments in space and time. Therefore, Equations (3) and (4) can be transformed into the discrete expressions of Equations (6) and (7) after two one-dimensional arrays of *D_V_*(*j*) and *P*(*j*) are established.

(6) $$D_{V}(t)=D_{V}(j\Delta t)=D_{0}\exp[-\frac{E_{D}}{k(T_{0}-\beta j\Delta t)}]$$

(7) $$P(t)=P(j\Delta t)=\nu_{0}\exp[-\frac{E_{\varphi }-E_{0}}{k(T_{0}-\beta j\Delta t)}]$$

The partial derivative of *N_V_*(*r*,*t*) against *r* and *t* can be expressed as Equations (8) and (9) after discretization.

(8) $$\frac{\partial N_{V}(r,t)}{\partial t}=\frac{N_{V}(i,j+1)-N_{V}(i,j)}{\Delta t}$$

(9) $$\frac{\partial^{2}N_{V}(r,t)}{\partial r^{2}}=\frac{N_{V}(i+1,j)-2N_{V}(i,j)+N_{V}(i-1,j)}{{\left(\Delta r\right)}^{2}}$$

Thus, the diffusion equation of Equation (5) can be transformed into Equation (10) from which Equation (11) can be easily obtained.

(10) $$\frac{N_{V}(i,j+1)-N_{V}(i,j)}{\Delta t}=D_{V}(j)\frac{N_{V}(i+1,j)-2N_{V}(i,j)+N_{V}(i-1,j)}{{\left(\Delta r\right)}^{2}}-P(j)N_{V}(i,j)$$

(11) $$N_{V}(i,j+1)=D_{V}(j)\frac{N_{V}(i+1,j)-2N_{V}(i,j)+N_{V}(i-1,j)}{{\left(\Delta r\right)}^{2}}\Delta t+[1-P(j)\Delta t]N_{V}(i,j)$$

**Figure 2:**
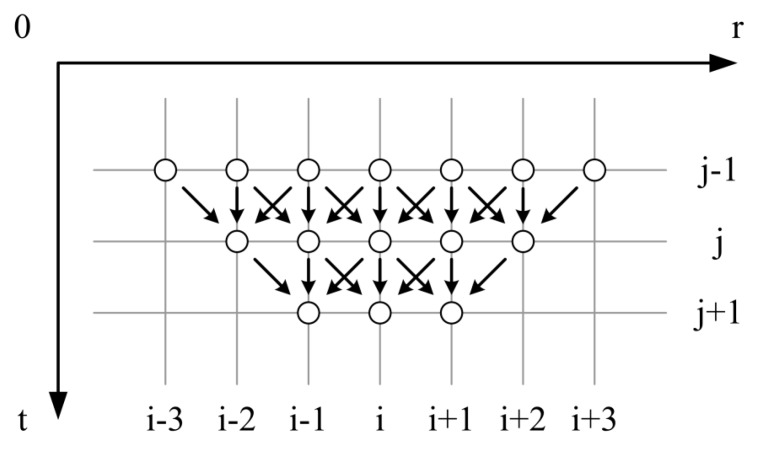
Schematic drawing of the calculation process of oxygen vacancy density in a semiconductor grain during the cooling process.

Equation (11) tells how the oxygen vacancy density at a point interacts with nearby positions after a time of Δ*t* elapses under the effects of diffusion and exclusion, which is shown in Figure 2. If *N_VS_* represents the *V_O_* density on the grain surface, the discrete initial condition of Equation (12) and boundary conditions of Equation (13) are considered.

(12) $$N_{V}=N_{VS},t=0$$

(13) $$\{\begin{array}{c} N_{V}(1,j)=N_{V}(2,j)\\ N_{V}(0,j)=N_{V}(2,j) \end{array}$$

It is noted that *N_VS_* is also a time-dependent variable once the cooling process starts. The density of oxygen vacancy on the grain surface would change when *V_O_* interacts with aerial oxygen, according to Equation (1). However, specific studies on this topic are still lacking. Therefore, a presumption is made that *N_VS_* is of linear dependence on time since *N_VS_*(*t*) = *N_VS_* × (1 + *k_VS_t*). In this scenario, *k_VS_* is a constant indicating the increasing rate of *V_O_* density on grain surface. Therefore, the numerical solution can be obtained by a MATLAB calculation, which provides possibilities to discuss the relationships among several important parameters that determine the gas-sensing characteristics of semiconductors.

As described by the Poisson’s law, the potential at grain boundaries is correlated to the space charge density in the depletion layer with the abrupt model applied [37], which is shown in Equation (14), provided that the oxygen vacancy is assumed to be first-order ionized.

(14) $$\frac{d^{2}V(x)}{dx^{2}}=\frac{q[N_{V}(x)]}{\varepsilon },0\leq x\leq w$$

A discrete one-dimensional array of *V*(*k*) is established by letting *V*(*x*) = *V*(*k*Δ*x*) where Δ*x* is the infinite small fragment in space. Thus, Equation (14) can be rewritten into the discrete expression of Equation (15).

(15) $$V(k-1)=\frac{qN_{V}(k)}{\varepsilon }{\left(\Delta x\right)}^{2}+2V(k)-V(k+1)$$

The boundary conditions can be expressed if *w* denotes the width of the depletion layer as Equation (16).

(16) $$\{\begin{array}{c} V(w)=0\\ \frac{dV(w)}{dx}=0 \end{array}$$

**Figure 3:**
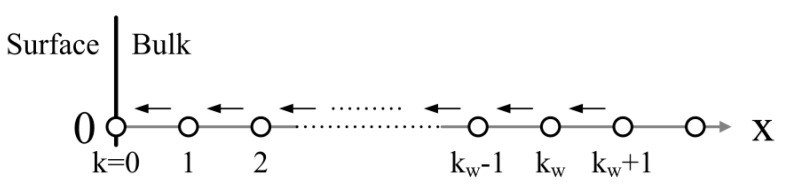
Schematic drawing of the calculation process of the potential barrier in the depletion layer of a semiconductor grain based on the density distribution of oxygen vacancies.

By using *k_w_* = *w*/Δ*x*, Equation (16) transforms to Equation (17), which is used to calculate the potential barrier height together with Equation (15) when *k* falls into [0, *k_w_*], as shown in Figure 3.

(17) $$\{\begin{array}{c} V(k_{w})=0\\ \frac{V(k_{w}+1)-V(k_{w})}{\Delta x}=0 \end{array}$$

The reduced resistance (*R*/*R*_0_) of the sensor can be calculated after the potential barrier height at the grain surface (*qV_S_*) is obtained by *qV_S_* = *qV*(0) (see Equation (18)).

(18) $$\frac{R}{R_{0}}=\exp(\frac{qV_{S}}{kT})$$

In this case, *R*_0_ is the flat-band resistance.

**Figure 4:**
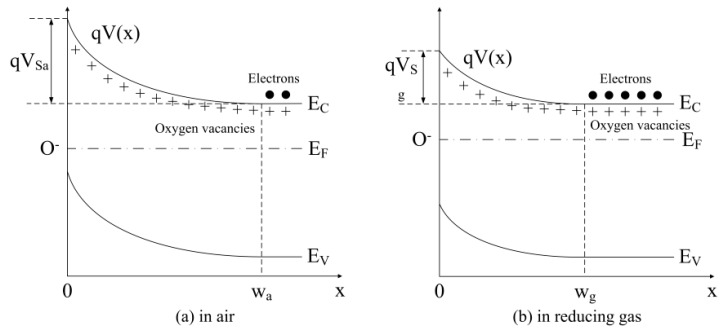
Potential barrier in the depletion layer of a semiconductor grain (**a**) in air and (**b**) in reducing gas.

The sensor response (*S*) is defined as the ratio of the resistance in air (*R_a_*) to the one in a reducing target gas (*R_g_*), as *S* = *R_a_*/*R_g_*. The response is stimulated by the target gas, which may consume the adsorbed oxygen on the grain surface and release electrons back to the depletion layer. This leads to a decrease in the depletion layer width. If *α* is introduced to indicate the concentration of the target gas, the change of the depletion layer width can be expressed by Equation (19) in the one-dimensional model [38], which is shown in Figure 4. Furthermore, *α* denotes the percentage of the seized electrons that released back to the depletion layer from the adsorbed oxygen.

(19) $$w_{g}=(1-\alpha )w_{a}$$

Therefore, the response (*S*) can be calculated based on the grain resistance before and after exposure to target gas. In the calculations, the parameters are set as follows: *D*_0_ = 0.0431 m^2^/s [39], *E_D_* = 2.7 eV [39], *ν*_0_ = 10^14^ s^−1^ [40], *E_D_* − *E_φ_* + *E*_0_ = 0.1 eV [34], *N_VS_* = 2 × 10^25^ m^−3^ [18], *k_VS_* = 3 × 10^−5^ s^−1^, *α* = 0.5, and *w* = 4 nm [41,42].

## 3. Results

### 3.1. Oxygen Vacancy Distribution

**Figure 5:**
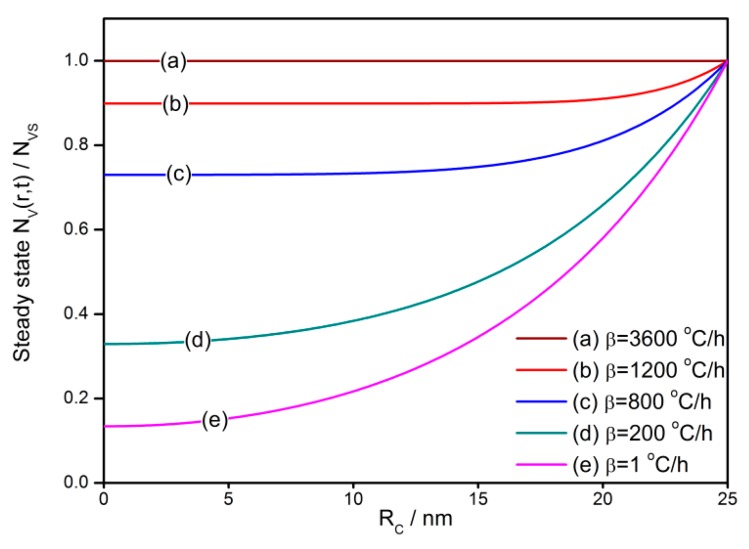
Influence of the cooling rate on the gradient distribution profile of oxygen vacancies in the semiconductor grain with a radius of 25 nm.

The influence of the cooling rate on the gradient distribution profile of oxygen vacancies in a 25 nm semiconductor grain is shown in Figure 5. The oxygen vacancies have various distribution profiles, which are determined by the cooling rate. For the quenched sample with the largest cooling rate of 3600 °C/h, the initial oxygen vacancies do not get sufficient time to migrate. Therefore, they are frozen at the place where they are at the starting of the cooling process, which leaves an almost uniform distribution throughout the grain. For the slowly-cooled sample, the initial oxygen vacancies migrate under the effect of diffusion and exclusion, which forms a gradient distribution in the grain. The gradient is of negative dependence on the cooling rate.

**Figure 6:**
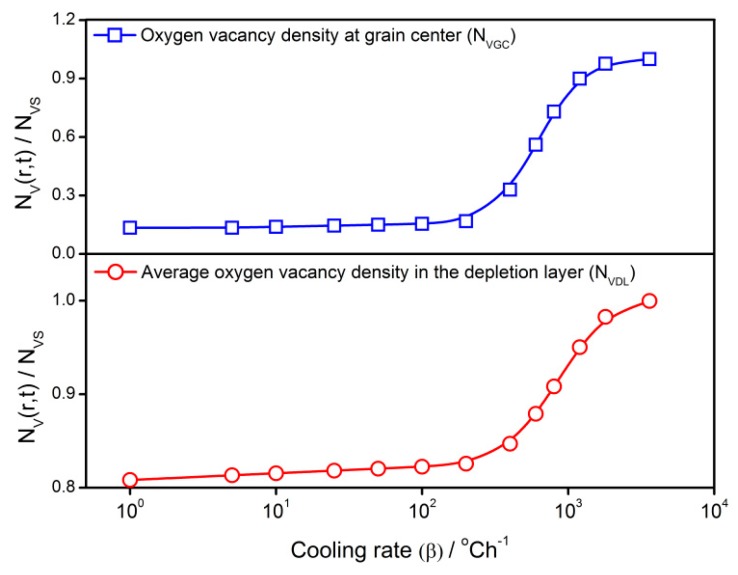
Influence of cooling rate on steady state oxygen vacancy density at the grain center (*N_VGC_*) and the average oxygen vacancy density in the depletion layer (*N_VDL_*) of a 25 nm grain.

Figure 6 shows the influence of the cooling rate on the steady state oxygen vacancy density at the center of the grain (*N_VGC_*) where the defect density is the smallest throughout the grain. It controls the density difference between the surface and center. The influence of the cooling rate on the average oxygen vacancy density in the depletion layer (*N_VDL_*) is also indicated in Figure 6. Within the cooling rate concerned, there are two regions where the vacancy densities have the linear correlation with the cooling rate. One is *β* = 1–200 °C/h and the other is *β* = 400–1000 °C/h. It infers that the *V_O_* gradient and the amount in the depletion layer can be easily controlled by the cooling rate in the two regions above. Considering the determination of the oxygen vacancy amount in the depletion layer on the sensor performances, it is possible to control the sensor properties by adjusting the cooling rate in the fabrication process.

**Figure 7:**
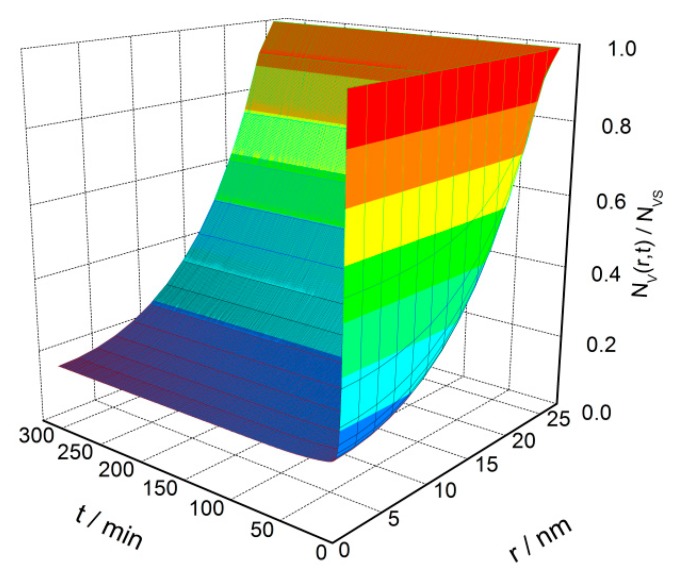
Time-dependent oxygen vacancy distribution in a 25 nm semiconductor grain from the initial uniform distribution to a steady state gradient distribution.

The transient distribution of oxygen vacancies in a semiconductor grain during the cooling process is illustrated in Figure 7, according to the numerical solution of Equation (5) in which *R_C_* = 25 nm and *β* = 100 °C/h. The initial uniform *V_O_* distribution is driven to distribute in a gradient profile. It describes that the *V_O_* distribution experiences three stages in the cooling process: (1) at the starting stage, the *V_O_* density is uniform throughout the grain, (2) then *V_O_* migrates under the effects of diffusion and exclusion at the transient stage, and, (3) lastly, the distribution reaches the steady state in a gradient profile.

### 3.2. Gas-Sensing Characteristics

**Figure 8:**
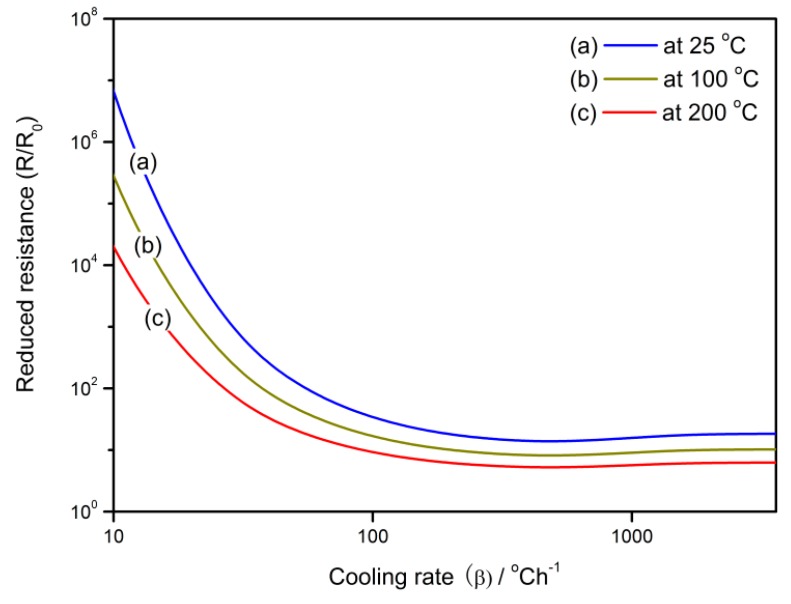
The relationship between reduced resistance (*R*/*R*_0_) of the gas sensor and the cooling rate at the operating temperatures of 25, 100, and 200 °C.

**Figure 9:**
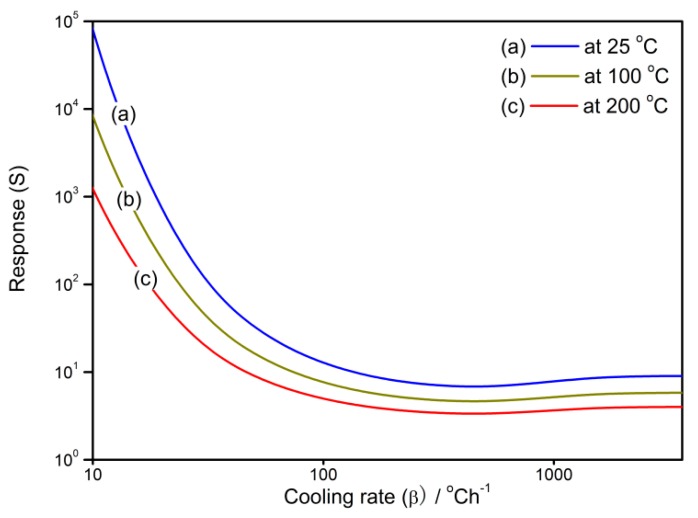
The relationship between the sensor response to reducing gas and the cooling rate at the operating temperatures of 25, 100, and 200 °C.

The relationships between gas-sensing characteristics and cooling rate at various operating temperatures are shown in Figure 8 and Figure 9 in which the reduced resistance (*R*/*R*_0_) and response (*S*) show similar performances. They descend with a cooling rate at the beginning and then reach the lowest point at the cooling rate of 400 °C/h. After that, the slight increases are observed until the sensor properties remain constant when the cooling rate is above 1800 °C/h.

**Figure 10:**
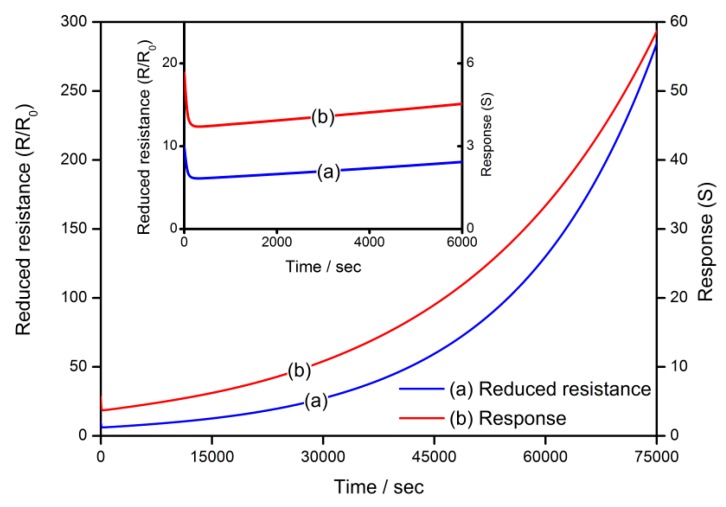
Transient states of gas-sensing characteristics of a semiconductor gas sensor during the cooling process.

The transient states of the gas-sensing characteristics of the semiconductor gas sensor during the cooling process are simulated in Figure 10. Both of the reduced resistance and response to reducing gas are increasing during the cooling process with the *V_O_* distribution driven from a uniform distribution to a gradient one, as described in Figure 5. However, sharp declines are observed at the start of the cooling process.

**Figure 11:**
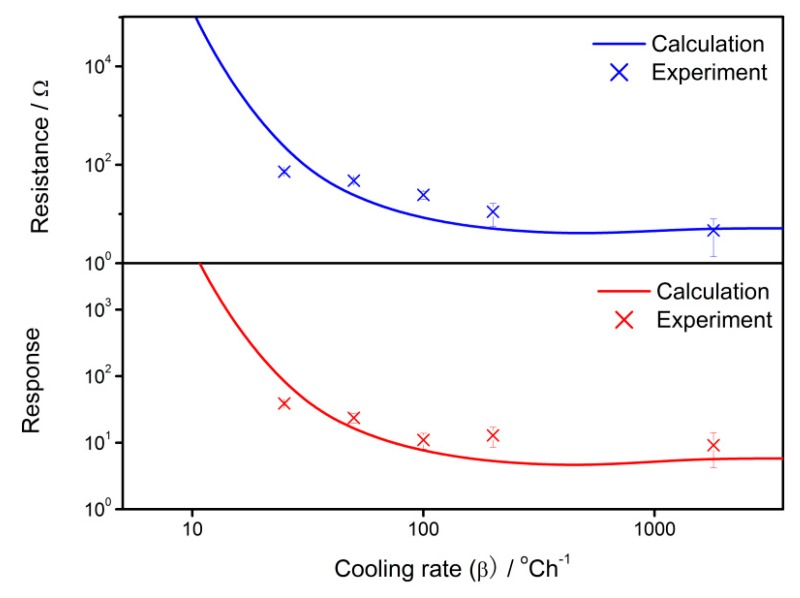
Calculation and experimental results of the sensor properties against the cooling rate of 25–1800 °C/h.

The calculation results are compared with the experimental results in order to validate the applicability of the gradient-distributed oxygen vacancy model. The experimental sensor resistance and response are introduced from the previous study [34], which describes the preparation details of the SnO_2_ thin film gas sensors and its dependence of sensing performances on the cooling rate in the fabrication process. The actual results are plotted in Figure 11, which also illustrates the calculated influence of the cooling rate on sensor properties. In this case, *R*_0_ is assumed to be 0.5. It is observed that the experimental plots located around the calculated correlations show good agreement. Therefore, the calculation in the present work has the potential to interpret the gas-sensing mechanism of semiconductor gas sensors.

### 3.3. The Grain Size Effects

**Figure 12:**
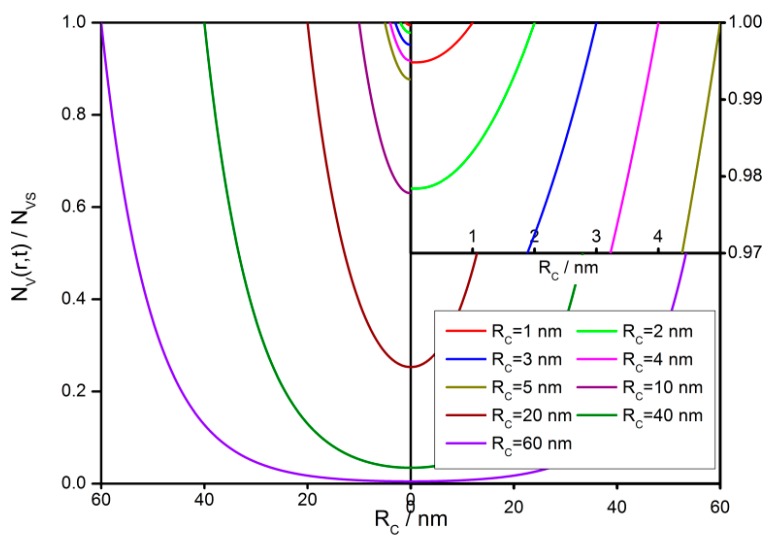
Steady-state distribution of oxygen vacancies in semiconductor grains with a radius of 1 to 60 nm.

The steady-state distributions of oxygen vacancies in the semiconductor grains with various grain sizes from 1 to 60 nm are illustrated in Figure 12. The distribution profiles show a significant grain size effect. In a 60 nm grain, the density difference is rather large between the surface and the center. However, less than 1% difference in *V_O_* density is found in a grain with *R_C_* = 1 nm. This means that a large grain can maintain a large internal *V_O_* density difference.

**Figure 13:**
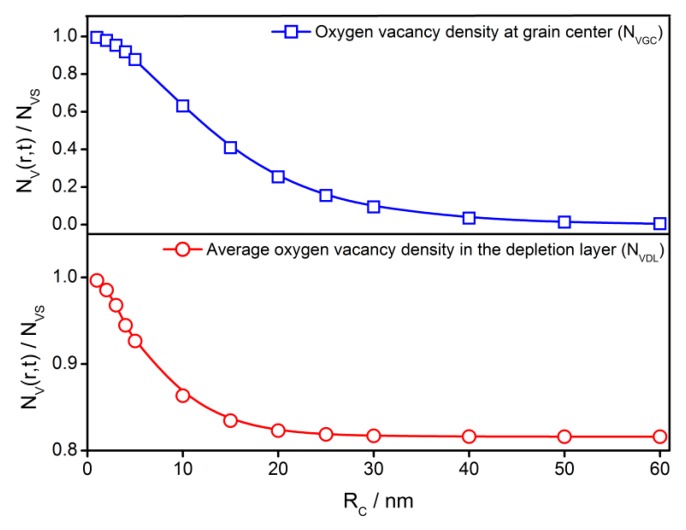
The grain size effect on the steady-state oxygen vacancy density at the grain center (*N_VGC_*) and average steady-state oxygen vacancy density in the depletion layer (*N_VDL_*).

The grain size also makes impacts on *N_VGC_* and *N_VDL_*. As shown in Figure 13, both *N_VGC_* and *N_VDL_* are of negative dependence with grain radius. The *V_O_* density at the grain center indicates the gradient of distribution profile. The *V_O_* amount in the depletion layer controls the supply of electrons, which results from the ionization of *V_O_*. It is concluded that the supply of the electrons in the depletion layer determines the amount of adsorbed oxygen on the grain surface and, therefore, decides the gas-sensing characteristics [18,25]. Figure 13 shows that *N_VDL_* keeps almost constant when the grain radius is larger than 30 nm. However, it increases with the reducing grain size when *R_C_* < 30 nm. This will lead to the grain size effects of the sensing performances of semiconductor gas sensors.

**Figure 14:**
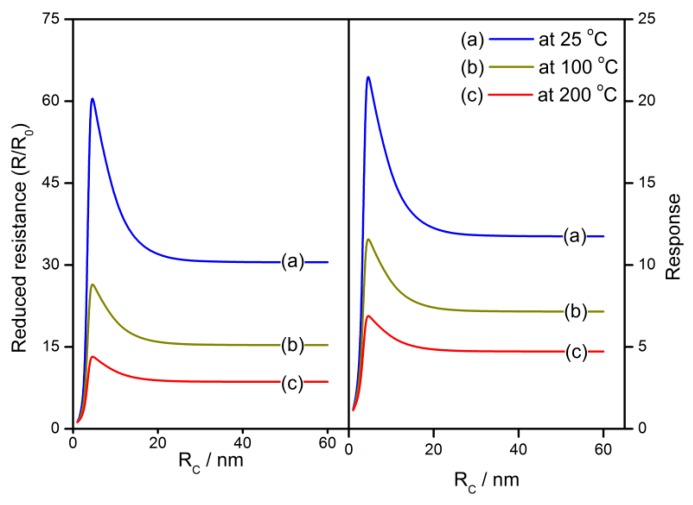
The grain size effects of reduced resistance and response at various operating temperatures of 25–200 °C.

**Figure 15:**
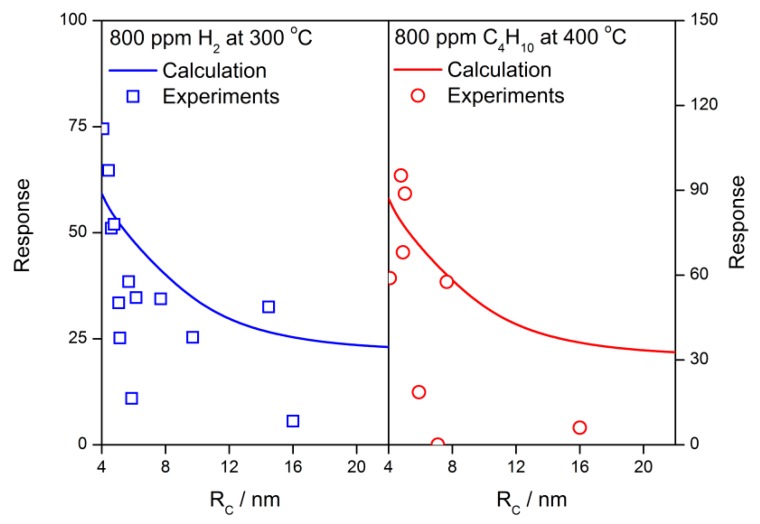
The grain size effects of calculation results and experimental response of the SnO_2_ gas sensors to 800 ppm H_2_ at 300 °C and 800 ppm C_4_H_10_ at 400 °C, which are extracted from C. Xu’s work [43].

The grain size effects of calculated sensor properties at various operating temperatures are shown in Figure 14. When the grain radius is approaching the depletion layer width, both of the reduced resistance and response to reducing gas have significant grain size effects that are in agreement with the experimental observation [20,43]. The comparison between calculation results and experimental responses is shown in Figure 15. The experimental plots are extracted from C. Xu’s report [43] while the calculation is completed by using the following parameter settings: *N_VS_* = 5 × 10^25^ m^−3^, *α* = 0.27 (H_2_), and 0.37 (C_4_H_10_) [42] and *T* = 300 °C (H_2_) and 400 °C (C_4_H_10_). The experimental grain size effects are well described by the calculation results. Furthermore, if the grain radius is equal to or smaller than the depletion layer width, the grain falls into volume depletion. In this case, both the reduced resistance and response decrease dramatically with the descending grain size until they reach unit when *R_C_* = 1 nm. For a volume-depleted grain, there are few free electrons inside it. The flat-band resistance *R*_0_ is rather large because of the descending of the Fermi level, which results in *R*/*R*_0_ to approach 1. On the other hand, the shortage of the electron supply prevents the adsorption of oxygen on the grain surface, which intercepts the gas-sensing mechanism. Therefore, a tiny calculated response is obtained. It infers that optimized gas-sensing properties could be obtained when the grain radius equals the depletion layer width by either controlling the grain size or adjusting the depletion layer width.

### 3.4. Re-Annealing Effect

**Figure 16:**
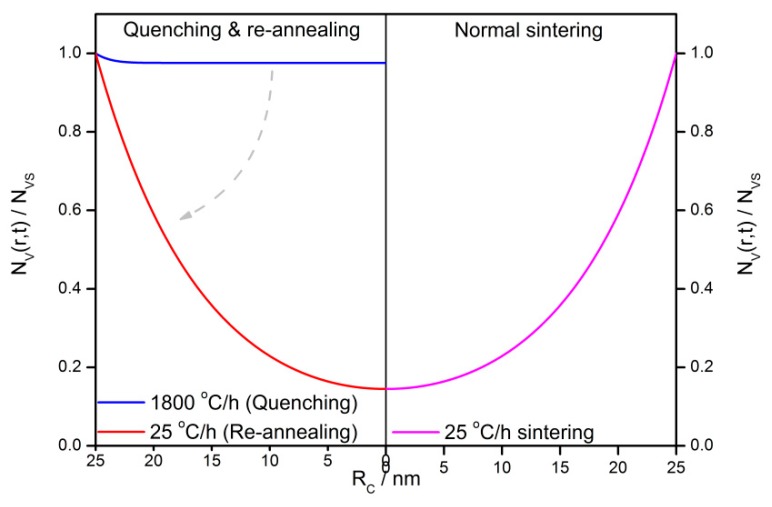
The steady-state oxygen vacancy distribution profiles in a 25-nm grain with normal sintering (*β* = 25 °C/h), quenching (*β* = 1800 °C/h), and re-annealing (*β* = 25 °C/h).

The re-annealing effect is found in the previous study and it mentions that the quenched grain would have the same property as the normal sintered grain after a second annealing is conducted [33]. Figure 16 shows the calculated *V_O_* distribution profiles in semiconductor grains with the radius of 25 nm. The quenched grain with a cooling rate of 1800 °C/h has an approximately uniform *V_O_* distribution. After that, a re-annealing with a cooling rate of 25 °C/h is conducted and a gradient *V_O_* distribution is obtained. The result is the same as the one in the normal sintered grain with a cooling rate of 25 °C/h. It infers that the *V_O_* migration is frozen by quenching and restarted by re-annealing.

**Figure 17:**
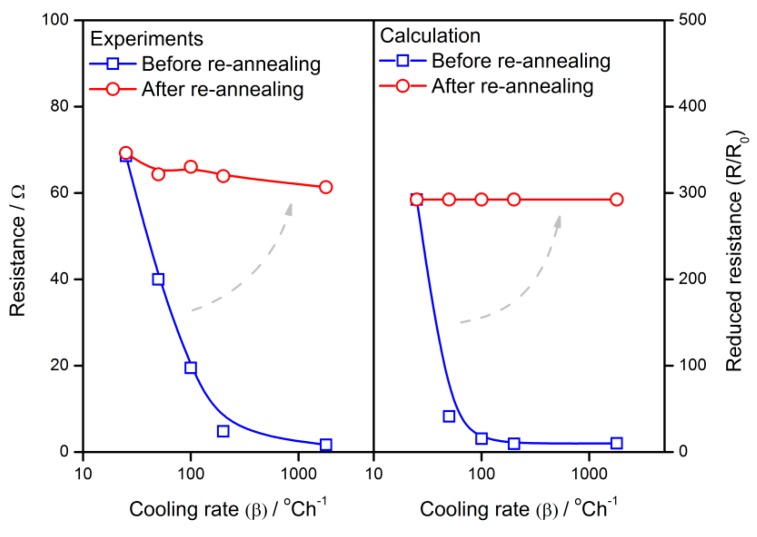
The re-annealing effect of experimental and calculated resistance of semiconductor gas sensors.

**Figure 18:**
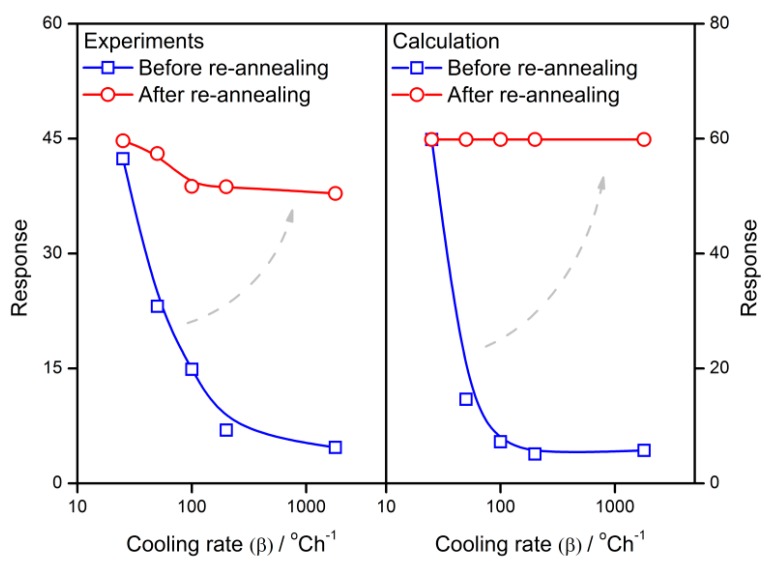
The re-annealing effect of experimental and calculated response of semiconductor gas sensors.

As shown in Figure 17 and Figure 18, after sintered with various cooling rates from 25 to 1800 °C/h, the grains are treated by a re-annealing process with a cooling rate of 25 °C/h. No matter what the cooling rates are used with the grain during the first time, the re-annealing process will lift the resistance and response to almost the same level as the one with a cooling rate of 25 °C/h. Although the experimental gas sensor properties appear as non-ideal plots when compared with the calculation, both of them show a similar tendency of the re-annealing effect.

### 3.5. Controlled Temperature Interval

**Figure 19:**
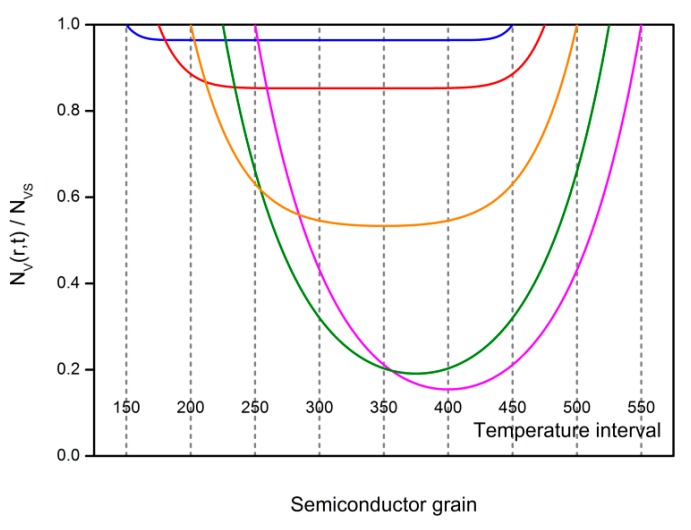
Oxygen vacancy distribution profiles in semiconductor grains with the radius of 25 nm are fabricated by using designed cooling processes, which have the same temperature difference of 300 °C but various starting and end temperatures.

**Figure 20:**
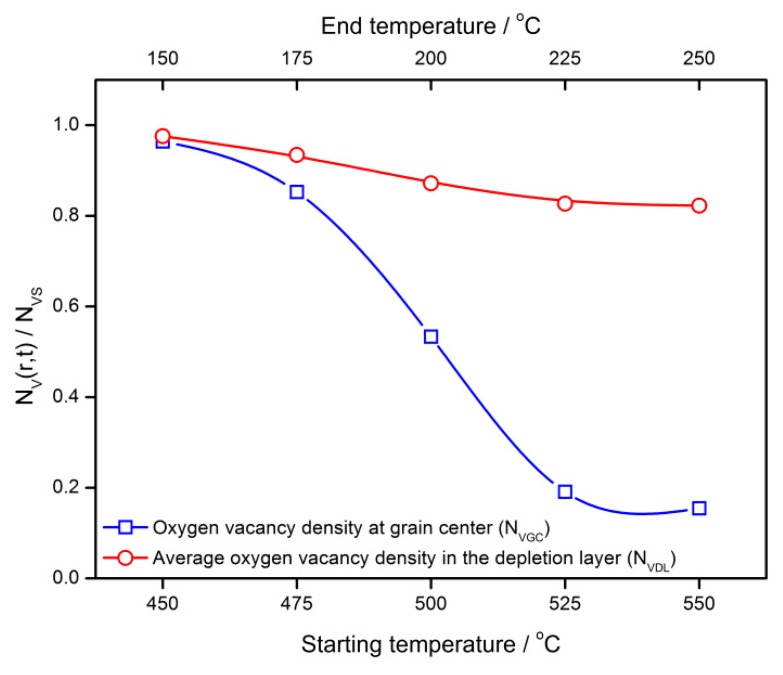
Influences of the temperature interval on the steady state oxygen vacancy density at the grain center (*N_VGC_*) and an average steady state oxygen vacancy density in the depletion layer (*N_VDL_*).

**Figure 21:**
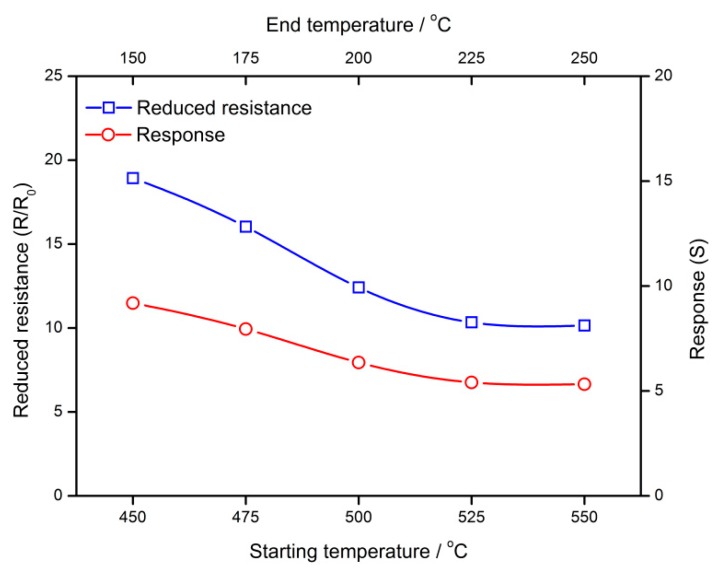
Influences of the temperature interval on gas-sensing characteristics of semiconductor gas sensors.

The results above show that the temperature setting in the cooling process may make impacts on the *V_O_* distribution as well as gas-sensing characteristics of semiconductor gas sensors. Thus, a series of designed cooling processes are simulated in order to investigate the influence of the temperature interval on the gas sensors. The temperature difference of the cooling process is set to be 300 °C while the starting and end temperatures are controlled, which is shown in Figure 19. The cooling rate is set to be 100 °C/h. In a semiconductor grain with the radius of 25 nm, the distribution profiles are dependent on the temperature interval. High temperature would drive *V_O_* to appear in a profile of a large gradient. However, after treatment in a low temperature, the grain has a uniform *V_O_* distribution in the center and gradient distribution near the surface. Figure 20 shows the influences of the temperature interval on *N_VGC_* and *N_VDL_*. Furthermore, the temperature interval can be used to control the gas-sensing characteristics of the semiconductor gas sensors. Figure 21 illustrates the dependence of reduced resistance and response on the temperature intervals. Both of the gas-sensing properties benefit from the low temperature treatment.

## 4. Discussion

In the sections above, the numerical analysis was carried out to find the numerical solutions of the diffusion equation of *V_O_* in the semiconductor grain. The solutions were used to illustrate the *V_O_* distribution and also to simulate the gas-sensing properties. The experimental sensor resistance and response were employed to check the validity of the calculation results, which shows good agreement. However, some considerations need to be discussed.

The gradient-distributed oxygen vacancy model was proposed to explain the influence of the cooling rate on the gas-sensing characteristics of semiconductor gas sensor. A diffusion equation of *V_O_* migration was established in a one-dimensional model based on the diffusion and exclusion effects. In the previous studies [33,34,44], the parameter of *T_E_* (end temperature of cooling process) had to be used to find the analytical solutions of the diffusion equation. This approximation is helpful to mathematical conduction but leads to an inaccuracy in the calculation results. The model cannot describe the *V_O_* behaviors during the cooling process precisely. The imperfection drives us to implement the numerical analysis method in the research of semiconductor gas sensors. As described in Equation (5), the diffusion equation is able to simulate the actual cooling process in the gas sensor fabrication. The discrete expression of the diffusion equation is established to find the numerical solutions based on boundaries conditions from the initial condition to the steady state via a step-by-step process. The numerical solutions provide a precise description of the *V_O_* behaviors in the cooling process and the gas-sensing characteristics. Furthermore, the influences of the cooling rate on the gas sensor properties are concluded in this work. Compared with previous research [33,34,44], the present results provide a more precise description of *V_O_* behaviors during the cooling process. Therefore, it is possible to calculate the gas-sensing properties of the sensors prior to practical fabrication and the calculation results are beneficial for the design of sensor preparation.

In the calculations, there are several presumptions that need to be considered. One of them is the value of *N_VS_* and its time dependence. *N_VS_* indicates the *V_O_* density on the grain surface. It is a temperature-dependent parameter and is influenced by many other factors such as *V_O_* formation and annihilation, according to Equation (1), as well as diffusion and exclusion effects based on Equation (5). However, the research on *N_VS_* is still lacking. Thus, a presumption has to be made that *N_VS_* is of linear dependence on time. A modification could be made based on the specific studies on *N_VS_*. The second one is the tunneling effect, which is not taken into consideration in the present study. Tunneling may take place in the semiconductor inside a grain or between grains. It provides possibilities for oxygen atoms and electrons to migrate through the potential barrier. Therefore, the diffusion equation of Equation (5) and the Poisson equation of Equation (14) should be amended. The third presumption is that the present calculation uses the same flat-band resistance (*R*_0_) when the grain is in volume depletion. In this situation, the reduced resistance (*R*/*R*_0_) and response (*S*) decrease rapidly to unit because of the lowered Fermi level, which would lead to a change in *R*_0_. However, it is not mathematically included in the calculation. A quantitative study for a volume-depleted grain is expected.

Although there are some approximations in the calculation, the present work provides an opportunity to understand the gas-sensing mechanism of the semiconductor in a quantitative manner. The validity of the calculation is checked by experimental results. The proposed model has probable applications in the sensor design by simulating the fabrication process as well as calculating the sensor characteristics. Some modifications could be made to the model if the specific research studies are involved. Then, it can be used to simulate the actual sensor characteristics not only in the fabrication process but also in the working circumstance. Therefore, it is possible to interpret the gas-sensing mechanism of the semiconductor and also some experimental phenomena, which are not fully understood such as aging and the degeneration of sensors.

## 5. Conclusions

The behaviors of *V_O_* in the cooling process of semiconductor gas sensors are described by the diffusion equation based on the gradient-distributed oxygen vacancy model. The numerical analysis method is used to find the accurate solutions, which illustrate the *V_O_* migration, the time-dependent distributions, and their determination on the gas-sensing characteristics of sensors. Several conclusions have been drawn.

(a) The *V_O_* distribution profile in semiconductor grain is determined by the cooling rate in the cooling process. Quenching or fast cooling can freeze the oxygen defects at the place where they form, which may results in an almost uniform distribution. A sintering or re-annealing with a low cooling rate may lead to a gradient *V_O_* distribution. For the *V_O_* density at the grain center and average density in the depletion layer, there are two regions where the defects densities have linear correlations with the cooling rate.
(b) The gas-sensing characteristics of the semiconductor correlate to the cooling rate. Negative relationships are observed for the reduced resistance and response to reducing gas against the cooling rate below 400 °C/h. The transient states of the gas-sensing properties during the cooling process show that there are sharp declines of reduced resistance and response at the starting of the cooling process. A designed re-annealing process can adjust the properties of semi-conductor gas sensors.
(c) The *V_O_* distribution and gas sensor properties appear to have significant grain size effects. Large grains may maintain a great gradient of *V_O_* distribution. When the grain radius approaches the depletion layer width, the gas-sensing properties increase until they reach the maximum. After the grain is in volume depletion, the sensor loses its gas-sensing properties rapidly. The optimized gas-sensing performances appear when *R_C_* = *w*.
(d) The cooling process in the fabrication can be designed for controlling the sensor performances, which are dependent on the temperature interval. The sensor resistance and response benefit from the low temperature treatment, which reduces the *V_O_* distribution gradient in the semiconductor grain.

## References

[1] Seiyama T., Kato A., Fujiishi K., Nagatani M. (1962). A new detector for gaseous components using semiconductive thin films. Anal. Chem..

[2] Shuping G., Jing X., Jianqiao L., Dongxiang Z. (2008). Highly sensitive SnO_2_ thin film with low operating temperature prepared by sol-gel technique. Sens. Actuators B.

[3] Gong S., Liu J., Xia J., Quan L., Liu H., Zhou D. (2009). Gas sensing characteristics of SnO_2_ thin films and analyses of sensor response by the gas diffusion theory. Mater. Sci. Eng. B.

[4] Zhao J., Wu S., Liu J., Liu H., Gong S., Zhou D. (2010). Tin oxide thin films prepared by aerosol-assisted chemical vapor deposition and the characteristics on gas detection. Sens. Actuators B.

[5] Yu L., Guo F., Liu S., Yang B., Jiang Y., Qi L., Fan X. (2016). Both oxygen vacancies defects and porosity facilitated NO_2_ gas sensing response in 2D ZnO nanowalls at room temperature. J. Alloys Compd..

[6] Li Z., Qin W., Zhao W., Wu X. (2014). Synthesis of flower-like Al doped ZnO microstructures by hydrothermal process and analysis of their gas sensing properties. Funct. Mater. Lett..

[7] Zeng J., Hu M., Wang W., Chen H., Qin Y. (2012). NO_2_-sensing properties of porous WO_3_ gas sensor based on anodized sputtered tungsten thin film. Sens. Actuators B.

[8] Barsan N. (1994). Conduction models in gas-sensing SnO_2_ layers: Grain-size effects and ambient atmosphere influence. Sens. Actuators B.

[9] Liu H., Wu S., Gong S., Zhao J., Liu J., Zhou D. (2011). Nanocrystalline In_2_O_3_-SnO_2_ thick films for low-temperature hydrogen sulfide detection. Ceram. Int..

[10] Bârsan N., Hübner M., Weimar U. (2011). Conduction mechanisms in SnO_2_ based polycrystalline thick film gas sensors exposed to CO and H_2_ in different oxygen backgrounds. Sens. Actuators B.

[11] Liu J., Lu Y., Cui X., Geng Y., Jin G., Zhai Z. (2017). Gas-sensing properties and sensitivity promoting mechanism of Cu-added SnO_2_ thin films deposited by ultrasonic spray pyrolysis. Sens. Actuators B.

[12] Zhao X., Shi W., Mu H., Xie H., Liu F. (2016). Templated bicontinuous tin oxide thin film fabrication and the NO_2_ gas sensing. J. Alloys Compd..

[13] Lee Y., Huang H., Tan O., Tse M. (2008). Semiconductor gas sensor based on Pd-doped SnO_2_ nanorod thin films. Sens. Actuators B.

[14] Li M., Zhou D., Zhao J., Zheng Z., He J., Hu L., Xia Z., Tang J., Liu H. (2015). Resistive gas sensors based on colloidal quantum dot (CQD) solids for hydrogen sulfide detection. Sens. Actuators B.

[15] Yu H., Song Z., Liu Q., Ji X., Liu J., Xu S., Kan H., Zhang B., Liu J., Jiang J. (2017). Colloidal synthesis of tungsten oxide quantum dots for sensitive and selective H2S gas detection. Sens. Actuator B Chem..

[16] Zhang B., Li M., Song Z., Kan H., Yu H., Liu Q., Zhang G., Liu H. (2017). Sensitive H_2_S gas sensors employing colloidal zinc oxide quantum dots. Sens. Actuators B.

[17] Morrison S.R. (1987). Mechanism of semiconductor gas sensor operation. Sens. Actuators.

[18] Yamazoe N., Shimanoe K. (2008). Theory of power laws for semiconductor gas sensors. Sens. Actuators B.

[19] Yamazoe N., Shimanoe K. (2009). New perspectives of gas sensor technology. Sens. Actuators B.

[20] Tamaki J., Zhang Z., Fujimori K., Akiyama M., Harada T., Miura N., Yamazoe N. (1994). Grain-size effects in tungsten oxide-based sensor for nitrogen oxides. J. Electrochem. Soc..

[21] Yamazoe N., Shimanoe K. (2008). Roles of shape and size of component crystals in semiconductor gas sensors I. Response to oxygen. J. Electrochem. Soc..

[22] Yamazoe N., Shimanoe K. (2008). Roles of shape and size of component crystals in semiconductor gas sensors II. Response to NO_2_ and H_2_. J. Electrochem. Soc..

[23] Korotcenkov G., Brinzari V., Boris Y., Ivanov M., Schwank J., Morante J. (2003). Influence of surface Pd doping on gas sensing characteristics of SnO_2_ thin films deposited by spray pirolysis. Thin Solid Films.

[24] Gupta V., Mozumdar S., Chowdhuri A., Sreenivas K. (2005). Influence of CuO catalyst in the nanoscale range on SnO_2_ surface for H_2_S gas sensing applications. Pramana.

[25] Yamazoe N., Fuchigami J., Kishikawa M., Seiyama T. (1979). Interactions of tin oxide surface with O_2_, H_2_O and H_2_. Surf. Sci..

[26] Liu H., Gong S., Hu Y., Liu J., Zhou D. (2009). Properties and mechanism study of SnO_2_ nanocrystals for H_2_S thick-film sensors. Sens. Actuators B.

[27] Wu J., Huang Q., Zeng D., Zhang S., Yang L., Xia D., Xiong Z., Xie C. (2014). Al-doping induced formation of oxygen-vacancy for enhancing gas-sensing properties of SnO_2_ NTs by electrospinning. Sens. Actuators B.

[28] Zou C., Liang F., Xue S. (2015). Synthesis and oxygen vacancy related NO_2_ gas sensing properties of ZnO: Co nanorods arrays gown by a hydrothermal method. Appl. Surf. Sci..

[29] Morrison S.R. (1982). Semiconductor gas sensors. Sens. Actuators.

[30] Ge Y., Wei Z., Li Y., Qu J., Zu B., Dou X. (2017). Highly sensitive and rapid chemiresistive sensor towards trace nitro-explosive vapors based on oxygen vacancy-rich and defective crystallized In-doped ZnO. Sens. Actuators B.

[31] Liu J., Gao Y., Wu X., Jin G., Zhai Z., Liu H. (2017). Inhomogeneous oxygen vacancy distribution in semiconductor gas sensors: Formation, migration and determination on gas sensing characteristics. Sensors.

[32] Zhang C., Geng X., Li J., Luo Y., Lu P. (2017). Role of oxygen vacancy in tuning of optical, electrical and NO_2_ sensing properties of ZnO_1-X_ coatings at room temperature. Sens. Actuators B.

[33] Liu J., Gong S., Fu Q., Wang Y., Quan L., Deng Z., Chen B., Zhou D. (2010). Time-dependent oxygen vacancy distribution and gas sensing characteristics of tin oxide gas sensitive thin films. Sens. Actuators B.

[34] Liu J., Gong S., Quan L., Deng Z., Liu H., Zhou D. (2010). Influences of cooling rate on gas sensitive tin oxide thin films and a model of gradient distributed oxygen vacancies in SnO_2_ crystallites. Sens. Actuators B.

[35] Shimizu Y., Kobayashi N., Uedono A., Okada Y. (2005). Improvement of crystal quality of GaInNAs films grown by atomic hydrogen-assisted RF-MBE. J. Cryst. Growth.

[36] Zhang M., Lin C., Weng H., Scholz R., Gösele U. (1998). Defect distribution and evolution in He^+^ implanted Si studied by variable-energy positron beam. Thin Solid Films.

[37] Sze S.M., Ng K.K. (2006). Physics of Semiconductor Devices.

[38] Liu J., Zhai Z., Jin G., Li Y., Monica F.F., Liu X. (2015). Simulation of the grain size effect in gas-sensitive SnO_2_ thin films using the oxygen vacancy gradient distribution model. Electron. Mater. Lett..

[39] Maier J., Göpel W. (1988). Investigations of the bulk defect chemistry of polycrystalline tin (IV) oxide. J. Solid State Chem..

[40] Kittel C. (2004). Introduction to Solid State Physics.

[41] Liu J., Liu X., Zhai Z., Jin G., Jiang Q., Zhao Y., Luo C., Quan L. (2015). Evaluation of depletion layer width and gas-sensing properties of antimony-doped tin oxide thin film sensors. Sens. Actuators B.

[42] Liu J., Jin G., Zhai Z., Monica F.F., Liu X. (2015). Numeral description of grain size effects of tin oxide gas-sensitive elements and evaluation of depletion layer width. Electron. Mater. Lett..

[43] Xu C., Tamaki J., Miura N., Yamazoe N. (1991). Grain size effects on gas sensitivity of porous SnO_2_-based elements. Sens. Actuators B.

[44] Liu J., Lu Y., Cui X., Jin G., Zhai Z. (2016). Effect of depletion layer width on electrical properties of semiconductive thin film gas sensor: A numerical study based on the gradient-distributed oxygen vacancy model. Appl. Phys. A.

